# Structural health monitoring of metal-to-glass–ceramics penetration during thermal cycling aging using femto-laser inscribed FBG sensors

**DOI:** 10.1038/s41598-020-69282-7

**Published:** 2020-07-23

**Authors:** Zhichun Fan, Xingzhong Diao, Kangjia Hu, Yong Zhang, Zhiyong Huang, Yanbo Kang, He Yan

**Affiliations:** 1 Institute of Nuclear and New Energy Technology, Key Laboratory of Advanced Reactor Engineering and Safety of Ministry of Education, Collaborative Innovation Center for Advanced Nuclear Energy Technology, Tsinghua University, Beijing, 100084 China; 2 Institute of Nuclear and New Energy Technology, Beijing Key Laboratory of Fine Ceramics, Tsinghua University, Beijing, 100084 China

**Keywords:** Engineering, Mechanical engineering, Applied optics, Optical techniques, Materials science, Techniques and instrumentation

## Abstract

Maintaining the mechanical strength and hermetic reliability of metal-to-glass–ceramics electrical penetration assembly (MTGC-EPA) is a key concern for ensuring the pressure boundaries of nuclear power plants. The transient temperature change caused by power adjusting or accidents in High Temperature Reactor Pebble-bed Modules may affect the structural health of sealing glass–ceramics, even leading to radiation leakage. To evaluate whether the function could survive temperature variations during the service life, thermal cycling aging experiments were imposed to MTGC-EPA. A grating length-mismatched sensing method to monitor the residual strain, an important factor of glass–ceramics structural health, was demonstrated in real-time by femto-laser inscribed fiber Bragg grating (FBG) sensor during the curing process and thermal cycling aging. Scanning electron microscope (SEM) and leakage rate tests were carried out to obtain the comparisons of microstructure and hermeticity before and after the thermal cycling. The residual strain showed a slight growth trend with thermal cycles repetition and it persisted a high value (~ 4,000 με) reflected by both Bragg wavelength shift and spectrum shape. The grating length mismatched single FBG embedded in glass–ceramics was feasible to demodulate the temperature and strain simultaneously, and the embedded FBG method achieved the structural health monitoring of MTGC-EPA during thermal cycling aging with good accuracy and reliability. Combining with the results of SEM and leakage rate detecting, the structural health of MTGC-EPA was demonstrated to be capable to endure the severe thermal conditions in nuclear reactors.

## Introduction

**Figure 1:**
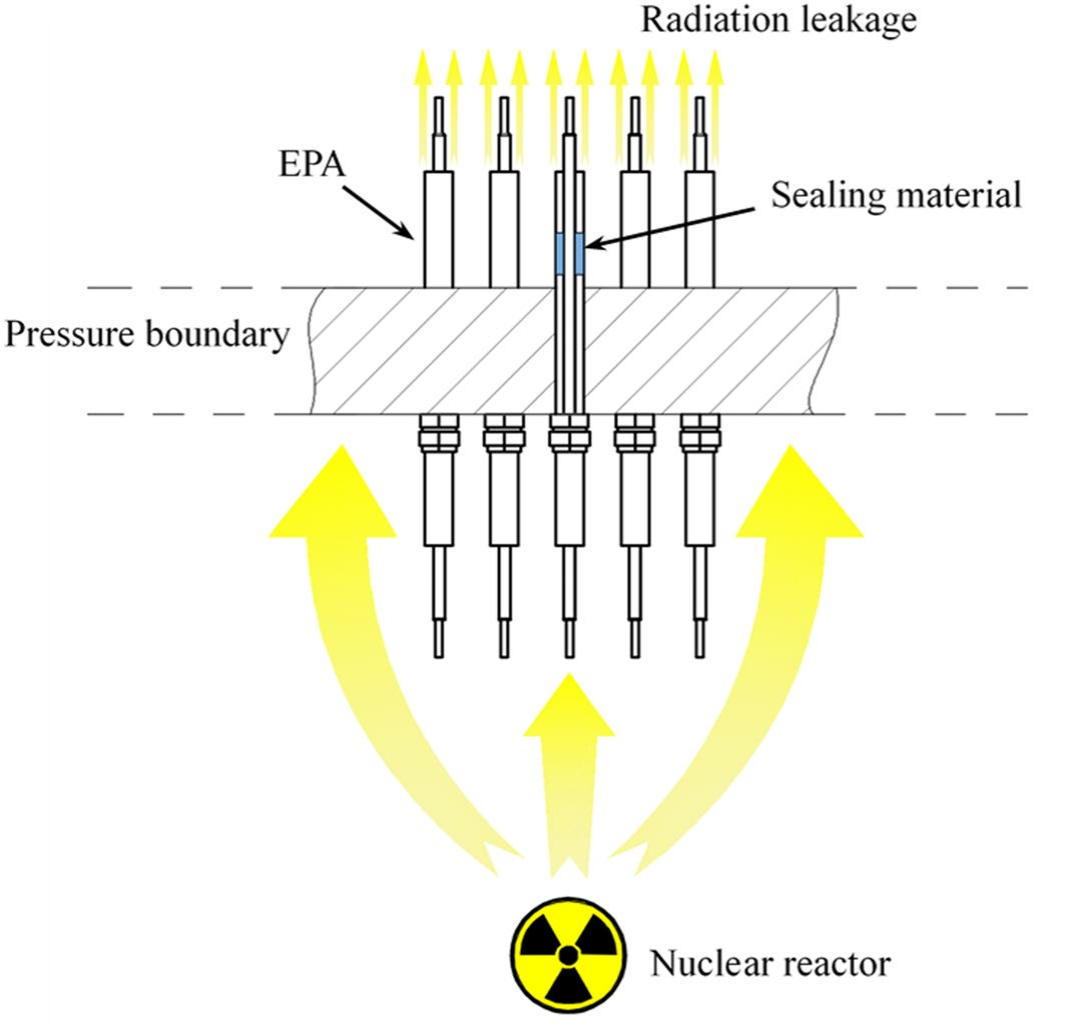
The schematic diagram of EPA installed on the pressure boundary of nuclear reactors.

Electrical penetration assembly (EPA) is the essential component to keep integrality of pressure boundaries in nuclear reactors as shown in Fig. 1. However, long-term service at high temperature in HTR-PM may decrease the structural strength and hermeticity of EPA. The failure of pressure boundaries may lead to severe disasters such as radiation leakage of Fukushima Daiichi Nuclear Power Plant, in which the leakage of hydrogen occurred at the penetrations and the gasket seals of the flange^1^. As a result, it is significant to ensure the hermetic reliability of EPA at high temperature environment.

Metal-to-glass seal structure EPA was widely used in nuclear reactors. This structure, consisting of steel shell, sealing glass and electric conductor, was designed according to the mismatched coefficient of thermal expansion (CTE)^2^. The difference between the CTE of steel shell and sealing material generated the residual strain in sealing material after the curing process, which maintained good hermetic reliability of EPA^3^. The similar CTE of sealing glass and conductor guaranteed the sealing glass will not be destroyed by the tensile stress resulted from the expansion of central conductor.

Glass–ceramics was proved to own superior performance than normal glass. It combines the general advantages of both crystalline ceramics and glass^4^. Glass–ceramics is a kind of high-softening-temperature-point glass, possessing better mechanical properties and higher temperature endurance, so it’s more appropriate to be applied in high temperature situations, compared with low-softening-point glass^5^. The residual strain in glass-to-metal seal structure was an important issue in the field of structural health monitoring (SHM) and it was difficult to measure directly due to the structure characteristics. Researchers have developed kinds of methods to detect residual strain, for example, Serbena et al.^6^ introduced the X-ray diffraction (XRD) and Raman spectroscopy were able to measure the residual strain in glass-to-metal seals. Lacondemine et al.^7^ and Buchheit et al.^8^ used indentation method to measure the stress in glass-to-metal seals, which was also destructive to the experimental model. Huntley et al.^9^ applied Photoluminescence spectroscopy (PLS) technique to achieve the non-destructive measurement of residual stress in glass-to-metal seals. However, all these approaches were unable to achieve the remote real-time on-line monitoring of glass-to-metal seals during its service life, especially in the harsh environment of nuclear power plants. Therefore, given that thermal variations happened in nuclear power plants and it would affect the structural health of metal-to-glass–ceramics sealing electrical penetration assembly (MTGC-EPA), it was necessary to develop a reliable method to achieve the remote on-line non-destructive SHM of MTGC-EPA, which could survive the thermal shocks during the harsh thermal variation conditions in nuclear power plants.

The fiber-optic monitoring system has been demonstrated to monitor multiple parameters (strain, temperature, deformation, etc.) successfully in the field of SHM^10–12^. Based on the previous explorations about EPA with low-softening-point glass, fiber Bragg grating (FBG) sensor was one of the most appropriate methodology to achieve the SHM of MTGC-EPA^13,14^, which was resistant to harsh environments and easily fused with glass material due to the similar components (SiO_2_).

In this research, the MTGC-EPA model was manufactured through a special heating process of glass–ceramics firstly. Before the manufacturing process, an optical fiber with FBG was embedded into the glass–ceramics to monitor the curing process and measure the residual strain. Considered the internal environment of nuclear reactors, high temperature and high dose radiation, the femto-laser inscribed FBG was applied in the experiment^15^. To examine whether MTGC-EPA could endure severe thermal variations, a long period thermal cycling aging was carried out, which was a comprehensive consideration for the combined cycling conditions in nuclear reactors (start-up, operation, shut-down, etc.) The applied thermal cycling aging was long-term continuous (more than 70 hours) and the temperature variations was intensive (22 cycles) and harsh (from 100 to 450 °C), which were more severe than the actual operating conditions (twice as large as safety margin). Those conditions were considered as strict assessments for both the structural health of MTGC-EPA and the capability of the fiber Bragg grating sensor. Compared with relevant researches^4,16^, the mechanical robusticity and hermetic reliability were initially evaluated in real time by the FBGs during such a long-term thermal cycling aging experiment with several shocks. Recent researches^17,18^ showed the temperature compensation method was applied widely to monitor the temperature and strain simultaneously, in this research, one single FBG embedded in sealing glass–ceramics was designed as length mismatched with sealing material to monitor the temperature and residual strain simultaneously based on the chirped spectrum. In addition, the scanning electron microscope (SEM) and the leakage rate detecting test were carried out to support the structural strength and hermetic reliability.

## Finite element model

**Figure 2:**
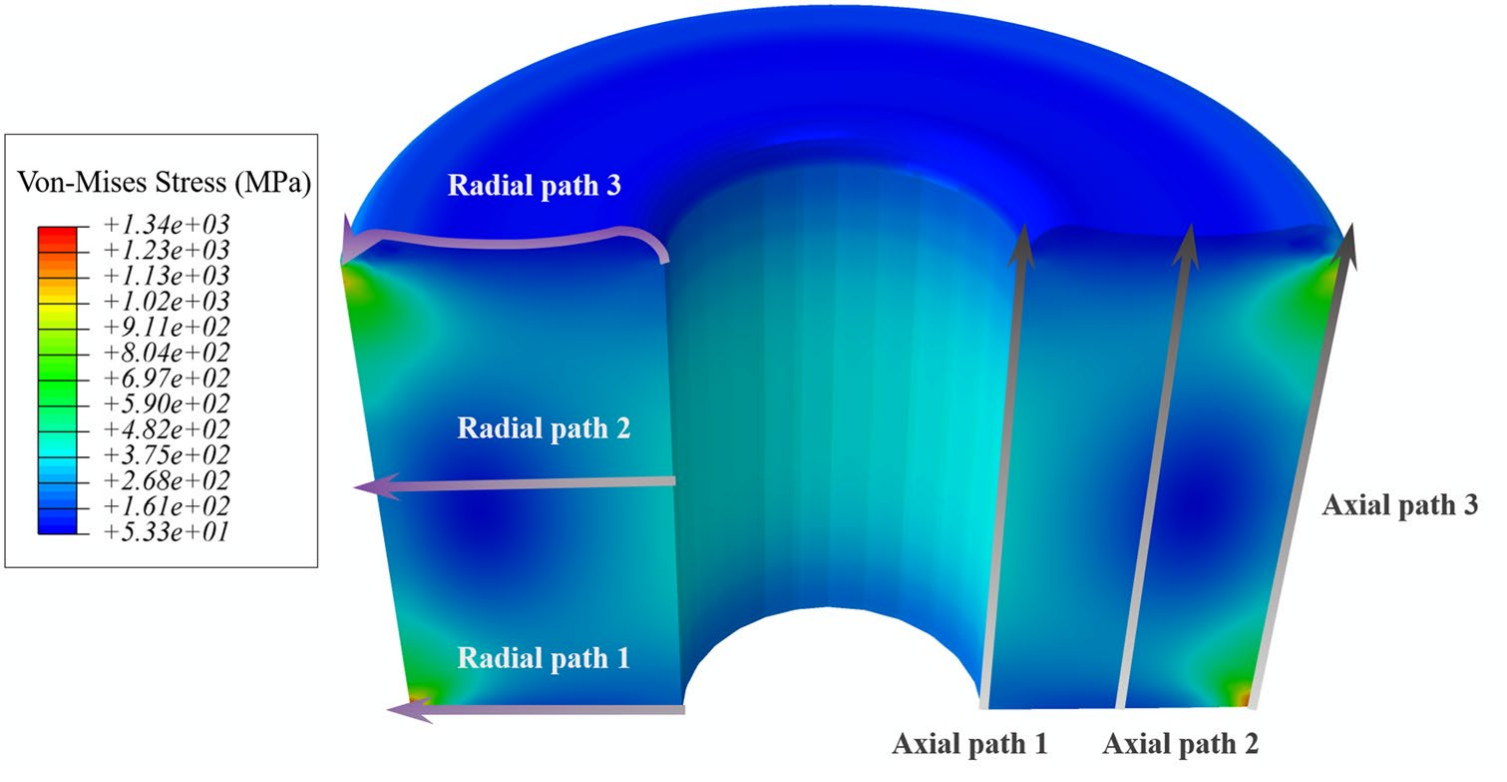
The stress contour of the cross section of glass–ceramics after curing process.

A finite element model of MTGC-EPA was built to predict the residual strain distribution in glass–ceramics after curing process and provide theoretical comparisons for the experimental monitoring results. The numerical simulation of residual strain in metal-to-glass structure has been demonstrated as a reliable method^19,20^. In this research, the linear-elastic model was applied because there was no obvious plastic deformation happened under the glass transformation temperature. The Young’s modulus and the CTE of each material were temperature related data. The top surface of glass–ceramics was concave to be consistent as the real model (see Fig. 2).

**Figure 3:**
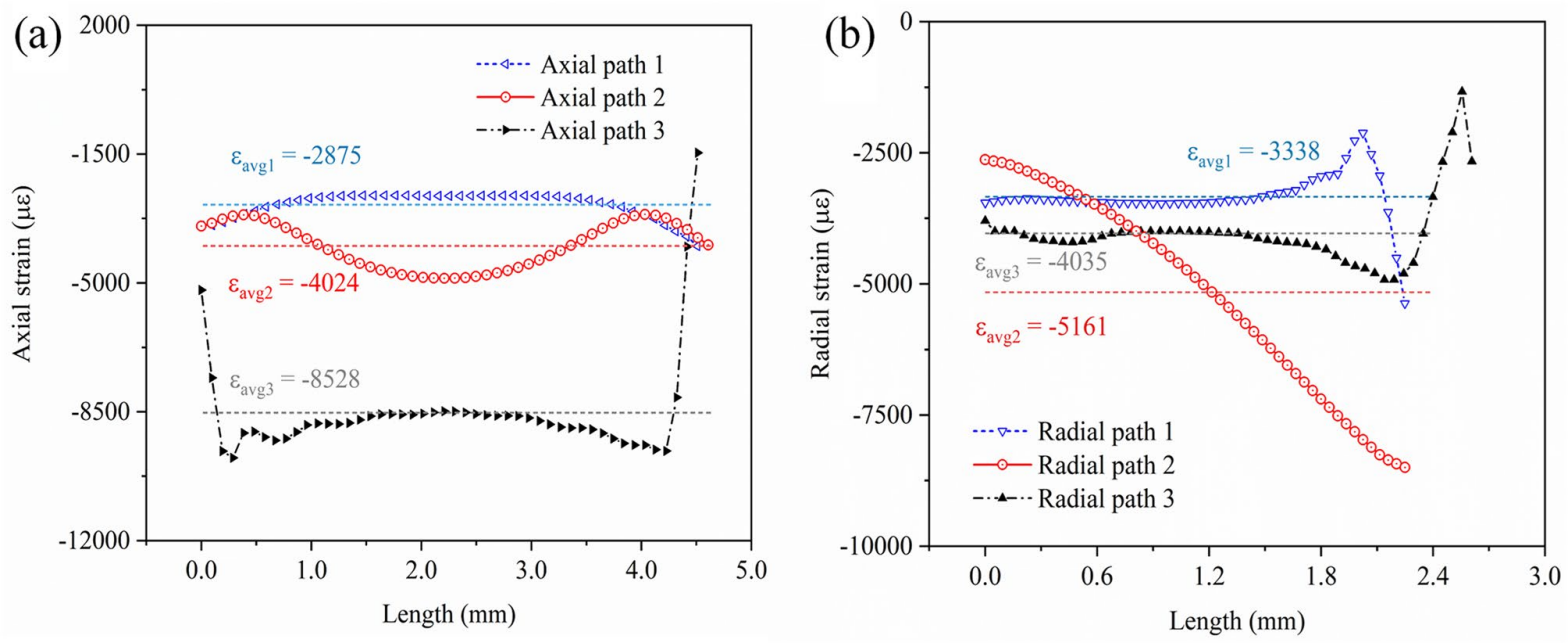
(**a**) The axial strain distribution along the three paths in glass–ceramics; (**b**) the radial strain distribution along the three paths in glass–ceramics.

As the simulated results showed, after curing process, the glass–ceramics was compressed by the outer metal shell, and the compressive stress dominated stress distribution. Nearly no tensile stress was identified, which was good for the mechanical strength, because the glass–ceramics could bear excellent compressive strength but poor tensile strength. To scrutinize the strain distribution inside the glass–ceramics quantitatively, the specific value was extracted along the 6 paths (3 for axial and 3 for radial) as shown in Fig. 3. The axial path 2 would be the exact location of embedded FBG sensor in glass–ceramics. The average axial strain increased from inside to outside of the glass–ceramics (Fig. 3a), because the compression induced by the difference of CTE of the steel shell/glass–ceramics. The average radial strain in the middle was larger than the top and bottom of glass–ceramics (Fig. 3b).

## Experimental design and setup

### FBG sensors

FBG sensor was proved to be sustained after long-term thermal aging^21^, so it was feasible to accomplish the SHM of composite structure during thermal cycling aging experiments. FBG owns a good sensitivity when measuring temeprature variation Δ*T* and strain *ε*, and the relationship between Bragg wavelength and measured parameters has been given by:

1 $$\frac{{\Delta \lambda _{{\text{B}}} }}{{\lambda {}_{{\text{B}}}}}{\text{ = }}(\zeta + \alpha ) \times \Delta {\text{T}}$$

2 $$\frac{{\Delta \lambda _{{\text{B}}} }}{{\lambda {}_{{\text{B}}}}} = \varepsilon _{1} - \left( {\frac{{n^{2} }}{2}} \right)[p_{{11}} \varepsilon _{t} + p_{{12}} (\varepsilon _{1} + \varepsilon _{t} )]$$

where Δλ_B_ is the wavelength shift induced by external changes, λ_B_ is the initial central wavelength, ζ is the thermo-optic coefficient, α is thermal expansion coefficient, p_ij_ is strain-optic coefficient, ε_1_ is the strain along fiber axis, ε_t_ is the strain transverse to fiber axis, and n is the effective refractive index.

To identify the applicability in high temperature annealing and thermal cycles of grating sensor, a comparison between Type I (UV process) and Type II (femto-laser inscribed) had been made under the same experimental embedding conditions. To monitor the high-level strain in glass–ceramics and endure the long-term harsh environment, the FEMTO*Plus* FBG with type II grating provided by FemtoFiberTec^22^ with low polarization (0–5 pm) and scattering loss (< 0.2 dB) was applied in this experiment. To validate the feasibility of FBG sensors at harsh environment, the long-term thermal aging test was carried out. The tested FBG sensors were both embedded in the glass–ceramics and tested at 450 °C for 70 h, of which the details were shown in Table 1.

**Table 1:** Two types of FBG used in thermal aging test.

Tested FBG	Normal FBG	FEMTO*Plus*
Grating type	Type I	Type II
Formation	UV photon absorption process	Threshold dependent multiphoton ionization process
Index change	10^−5^–10^−3^	10^−2^
Temperature applicability	500 °C	1000 °C
Thermal sensitivity (dλ/dT)	0.0118	0.0122
Strain sensitivity (dλ/dε)	0.001209	0.001205

**Figure 4:**
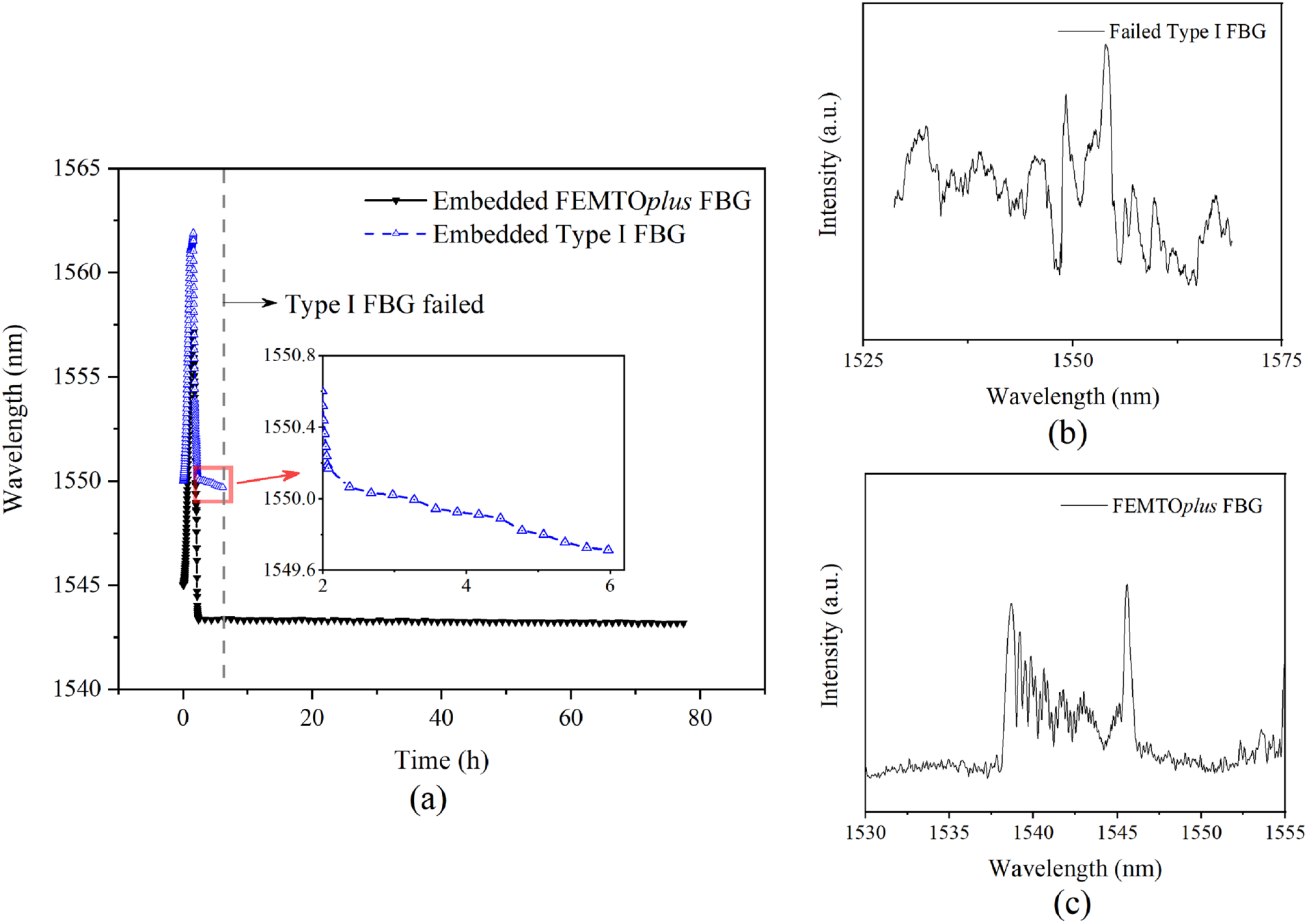
(**a**) The wavelength shift curve of the embedded FBGs in the aging test; (**b**) the spectrum of failed Type I FBG after thermal aging; (**c**) the spectrum of femto-laser inscribed FBG after thermal aging.

As shown in Fig. 4a, the FBGs were experiencing the same heating process at the beginning to be fused with glass–ceramics and then cooling to 450 °C. The normal FBG with Type I grating failed after 4-h aging at 450 °C. The spectrum and wavelength of failed FBG was hard to distinguish by the interrogator (provided by Gaussian Optics, Wuhan, China), from Fig. 4b. On the contrary, the femto-laser inscribed FBG remained a rational spectrum after long-term thermal aging (see Fig. 4c), and the Bragg wavelength shift of femto-laser inscribed FBG caused by the constant high temperature was less than 0.05 nm. These results thus demonstrated the feasibility of femto-laser inscribed FBG in the monitoring of MTGC-EPA during thermal cycling aging experiment.

### MTGC-EPA

**Figure 5:**
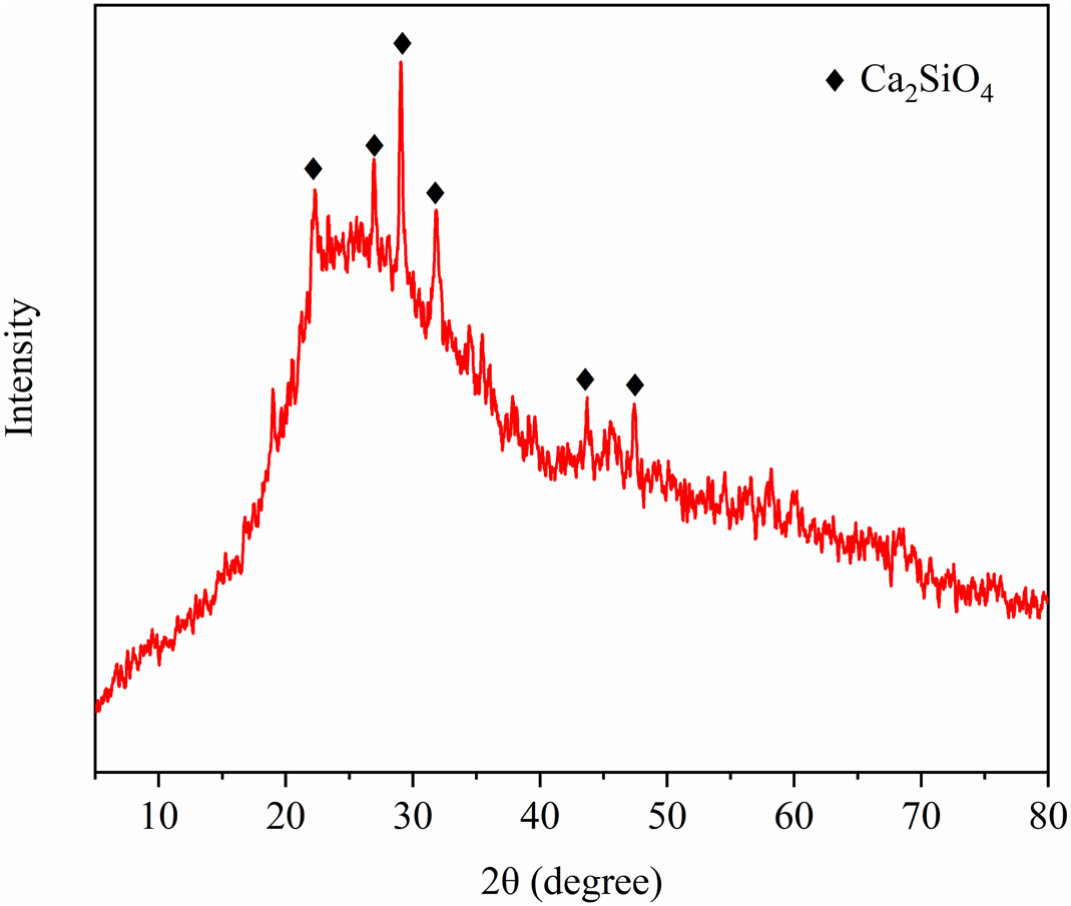
The XRD result of KF-Z glass–ceramics.

**Figure 6:**
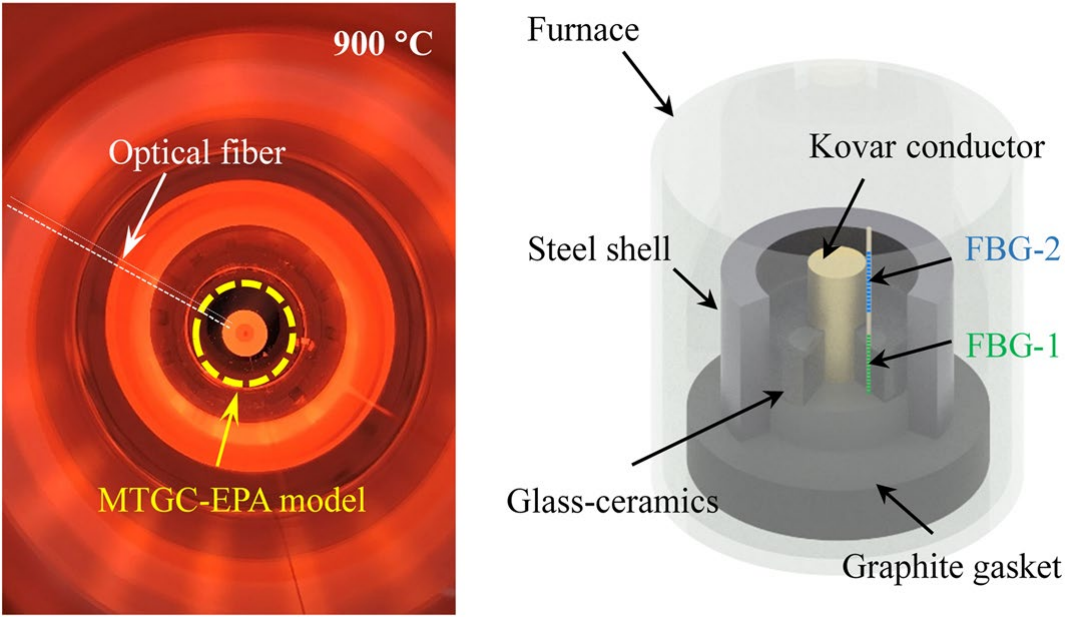
(**a**) Actual photograph of MTGC-EPA model at 900 °C; (**b**) the 3D model of MTGC-EPA model in heating furnace.

The glass–ceramics was prepared with paths for FBGs to monitor the residual stress. The steel shell, the sintered glass–ceramics, FBGs and conductor were fixed by a graphite packing, which would not stick to each other part of MTGC-EPA model. Main compositions of the glass–ceramics were shown in Table 2. Figure 5 showed the X-ray diffraction pattern (XRD) of the glass–ceramics, indicating that Ca_2_SiO_4_ crystalline phase existed in the glass–ceramics. The MTGC-EPA model was placed onto the silica gasket plate in the heating furnace to experience the manufacturing (see Fig. 6a) and thermal cycling aging. For validating the grating length mismatched sensing method and comparing with the temperature compensation method, an extra grating was placed close to the length mismatched embeded grating. Two grating regions (each for 12 mm) were inscribed on one fiber, and the central wavelength were respectively 1545 nm (FBG-1) and 1555 nm (FBG-2). During the experiment, FBG-1 was embedded in glass–ceramics to monitor the strain and temperature simultaneously, and FBG-2 was located nearby the glass–ceramics as the temperature compensation sensor. On one hand, the strain of glass–ceramics monitored by FBG-1 was demodulated by subtracting wavelength shift of FBG-2 from that of FBG-1. On the other hand, the strain of glass–ceramics also was demodulated via the spectrum analysis. The manufactured MTGC-EPA model with optical fiber sensors was shown in Fig. 6b.

**Table 2:** Compositions of KF-Z glass–ceramics.

Compound	Composition (mol%)
SiO_2_	60
Al_2_O_3_	8
B_2_O_3_	15

### Thermal cycling aging experiment

To examine whether the mechanical strenghth and hermetic reliability could bear the harsh environment of nuclear power plants, 22 thermal cycles were imposed to the EPA model. The thermal cycles rose from 100 °C (the normal working temperature) to 450 °C (2 times as the most extreme condition in HTR-PM) at 15 °C/min. Two different cooling rates (1.5 °C/min and 5 °C/min) were carried out in this research. The end of the fiber was connected to the interrogator through the FC connector, so the Bragg wavelength data and the spectrum of FBG would be obtained in real-time by the interrogator and supported software.

### SEM and pressure test

The parallel samples of MTGC-EPA model with embedded FBG sensor were obtained beofore and after thermal cycles for the SEM test. The micro-combination status of FBG and glass–ceramics were captured by SEM to examine the monitoring reliability of FBG sensor.

**Figure 7:**
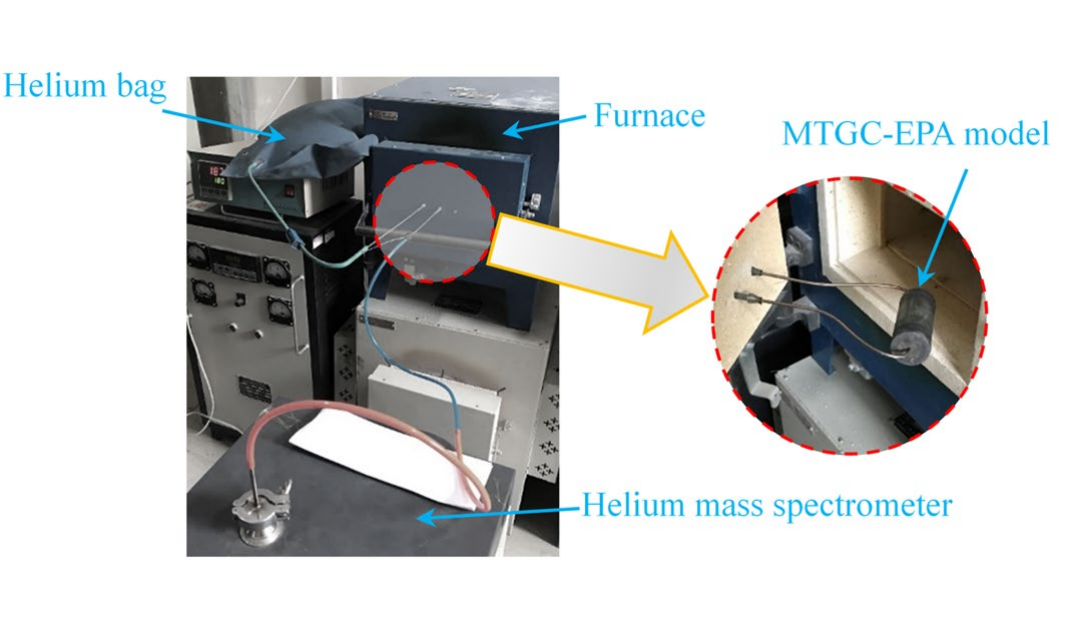
The experimental setup for the leakage rate detection test of MTGC-EPA model.

The leakage rate test was carried out to detect the leakage rate of MTGC-EPA model before and after thermal cycles. The leakage rate was a direct index to evaluate the hermetic failure of EPA. Both sides of the tested model were welded to a matched steel plate to form a vapor chamber as shown in Fig. 7. A pipeline was extended from the chamber to the helium mass spectrometer, and the other side of the chamber was connected to a helium bag. The tested model was placed into a heating furnace to make the leakage rated detected at combined high temperatures (100–450 °C) and vacuum environments.

## Results and discussion

### Curing process and residual strain monitoring

**Figure 8:**
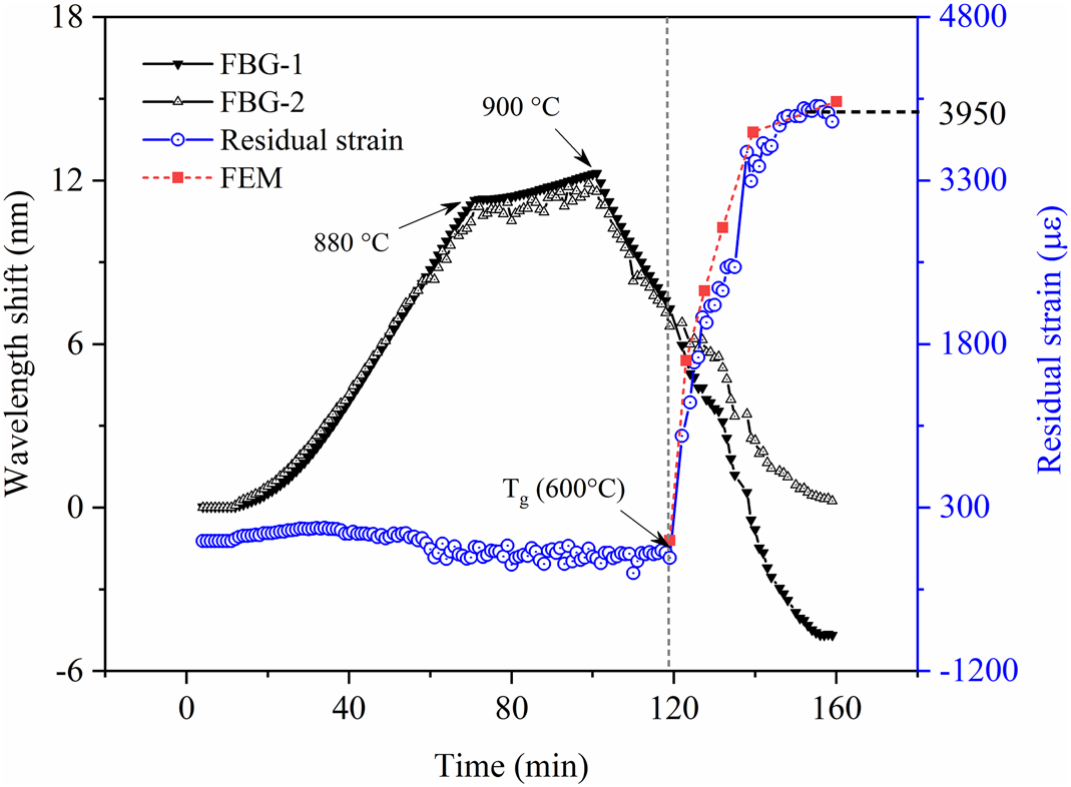
The monitored residual strain of FBGs during manufacuring process and corresponding FEM results.

By temperature compensation method, FBG-1 and FBG-2 achieved the real-time monitoring of temperature and residual strain during the curing process, as shown in Fig. 8. To avoid the FBG measuring deviation caused by the cross sensitivity of temperature and strain, researchers developed many techniques such as using special coating^23^, characterizing strain by bandwidth^24^, improving the demodulation method^25^ and so on. Setting temperature compensation sensor was a useful method to demodulate the temperature and strain^26^. The Bragg wavelength shift of FBG-1 represented the simultaneous change of temperature and residual strain, and FBG-2 only represented temperature change. As a result, the different value between them represented the residual strain change, illustrated by the blue line in Fig. 8. The glass transition temperature T_g_ after which the glass–ceramics would turn from the rubbery state to the vitreousness was detected. Based on the Eq. (2), the mean value of residual strain was estimated from the Bragg wavelength shift. The axial residual strain in glass–ceramics under the specific heating process was about 3,900 με.

Based on the finite element method (FEM) model in the “Finite element model” section, the residual strain at different temperature during the curing process was obtained as the red line shown in Fig. 8. The deviation between the monitored and numerical results was less than 5%, so the accuracy and reliability of this methodology were verified.

**Figure 9:**
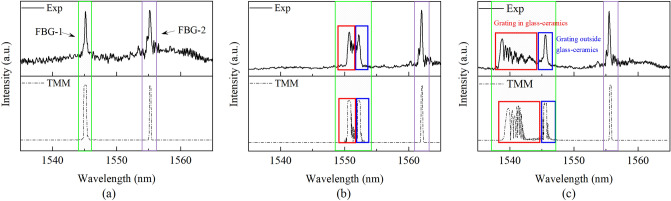
The experimental and simulated spectra of femto-laser inscribed FBG-1 and FBG-2 at different stages of curing process; (**a**) The origin spectra of FBGs before manufacturing; (**b**) The spectra of FBGs when the strain begins to form in glass–ceramics; (**c**) The spectra of FBGs after curing process.

The results by grating length mismatched sensing method were presented and discussed. Given that the stress in glass–ceramics was non-uniform and high level shown in Fig. 3a, the origin spectrum of FBG-1 (Fig. 9a) would generate obvious distortions (see Fig. 9b,c) due to the chirped grating period when the residual strain began to formed^27^, so the softening point of the glass–ceramics was precisely monitored. As the temperature decreased to 100 °C, the residual strain in glass–ceramics increased, and the broadening and distortions of FBG-1 were more recognizable shown in Fig. 9c. This phenomenon was simulated by the transfer matrix method (TMM)^28^ based on the coupled mode theory as shown in Fig. 9c. The strain imposed to the grating region was the same as the strain distritbution of axial path 2 obtained by the FEM in “Finite element model” section. The spectra change of both experimental and simulated results was consistent. From the comparison between the experimental and simulated results of Fig. 9, the deviation of central wavelength was less than 5%, and the deviation of full width at half meduim (FWHM) was less than 15%. The measuring accuracy was validated, and the minor difference of FBG-1 was tolerated, because the mesh density of TMM method was much higher than the accuracy of the interrogator. To improve the accuracy of strain measuring, the Bragg wavelength of FBG-1 was extracted by Gaussian fitting method^29^.

**Figure 10:**
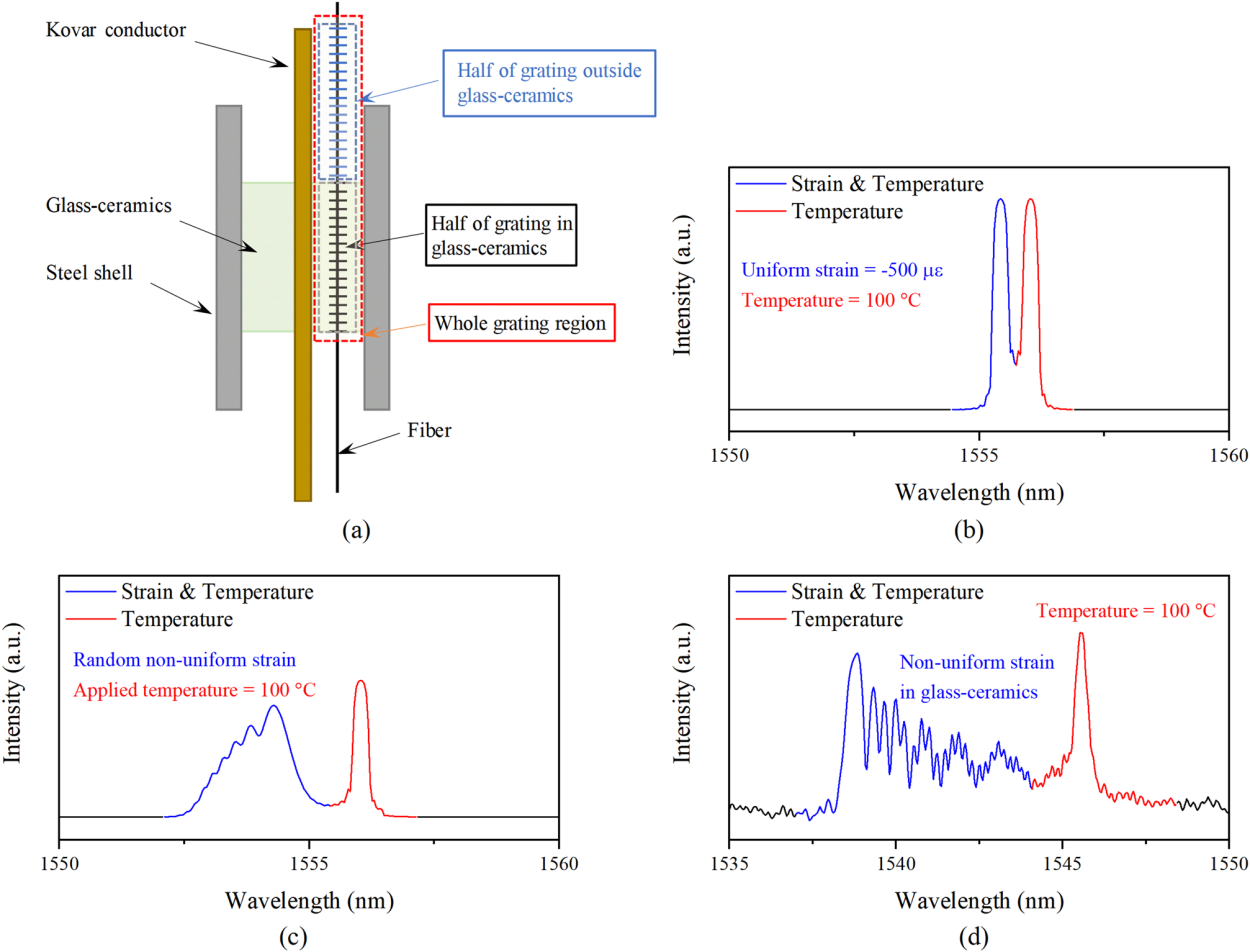
(**a**) The installation of length mismatched FBG in glass–ceramics; (**b**) The simulated spectrum of length mismatched FBG under applied uniform strain and 100 °C; (**c**) The simulated spectrum of length mismatched FBG under applied random non-uniform strain and 100 °C; (**d**) The experimental spectrum of length mismatched FBG undernon-uniform strain in glass–ceramics and 100 °C.

The length of FBG-1 (12 mm) was mismatched with the glass–ceramics (5 mm), so part of the FBG-1 would stretch out of the glass–ceramics without the influence of residual strain. Therefore, the spectrum of FBG-1 was divided into two sections^30^. One was the chirped section due to the non-uniform axial strain distribution in glass–ceramics as shown in the red box in Fig. 9c. The other was the distinct single peak at the right side of spectrum, which was only affected by the environmental temperature, marked by the blue box in Fig. 9. This phenomenon was proposed to be a feasible method to demodulate the temperature and strain by a single FBG sensor. As simulated by TMM, when the embedded grating length was about half of the whole grating region (Fig. 10a), the spectrum of the FBG would split into two peaks (Fig. 10b). The right peak only indicated the temperature of the measured model, and the left peak indicated both temperature and strain inside the model.

In our previous research, the FWHM of FBG was only affected by the non-uniform strain distribution, which was also presented in Fig. 10c. For the type of FBG applied in our research, the relationship between average strain distribution and the Bragg wavelength was almost linear^14^. The experimental FWHM of FBG embedded in glass–ceramics after curing process (see Fig. 10d) was about 4.02 nm, and the corresponding strain was about 3,500 με. Compared with the FEM results in section Finite element model, the deviation between the measured and theoretical results was less than 12%, and it was also consistent with the strain (3,900 με) calculated obtained from the temperature compensation method with deviation around 10%. For the temperature measurement, the Bragg wavelength shift of the right peak was in according with the relationship between wavelength and temperature. Therefore, the spectrum analysing of single FBG in glass–ceramics with mismatched length would achieve the simultaneous discrimination of temperature and strain with good accuracy and easier operations compared with temperature compensation method.

### Structural health monitoring of thermal cycling aging

**Figure 11:**
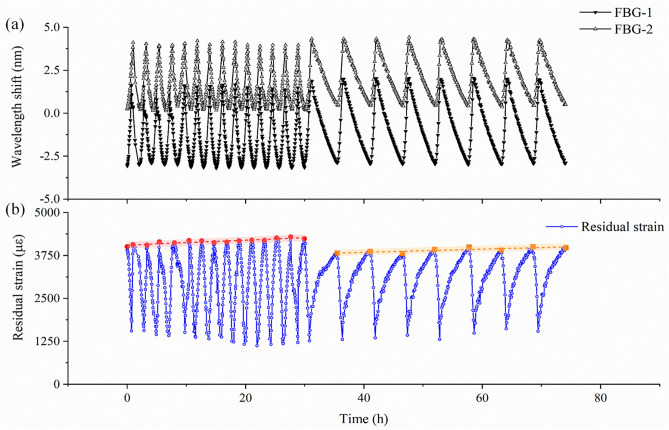
(**a**) The monitored wavelengths shift of FBGs during thermal cycling aging experiment; (**b**) the magnitude of residual strain monitored in thermal cycling aging.

The on-line monitoring results of 22 thermal cycles were shown in Fig. 11. The methodology based on femto-laser inscribed FBG was effective and the monitoring was consecutive in the thermal cycling aging. The monitored trend of residual strain was much more important than the exact magnitude. The cooling rate before 14th cycle (5 °C/min) was 2 times faster than that of 15th to 22nd cycles (1.5 °C/min). From the demodulated strain change in Fig. 11b extracted from Fig. 11a, the cooling rate introduced a small effect on the residual strain value. The strain would grow when the cooling rate was faster. Besides, the number of thermal cycles would give a insignificant positive correlation with the residual strain (less than 10%).

The influence factors of residual strain in glass–ceramics contained the crystallization properties (volume fraction, crystal shape, etc.) and the mechanical properties (Young’s modulus, CTE, etc.)^6^. The cooling rate before the softening point of glass–ceramics (> 600 °C) played an important role on the crystallization properties and the permanent strain value. Because the maximum temperature in thermal cycling was 450 °C and less than the T_g_ of glass–ceramics, the permanent strain wouldn’t generate distinct variations for this reason. Moreover, the enlarging of non-homogenous cooling rate would intensify the thermal gradient and strain rate of the MTGC-EPA. The residual strain reached a relatively constant value as the MTGC-EPA model was cycled further, and no severe strain relaxation of this model was observed in the thermal cycling aging. Hence, the appropriate amount of thermal cycles wouldn’t decrease the mechanical strength and lead to the stress relaxation in MTGC-EPA model, based on the monitoring result of temperature compensation method.

**Figure 12:**
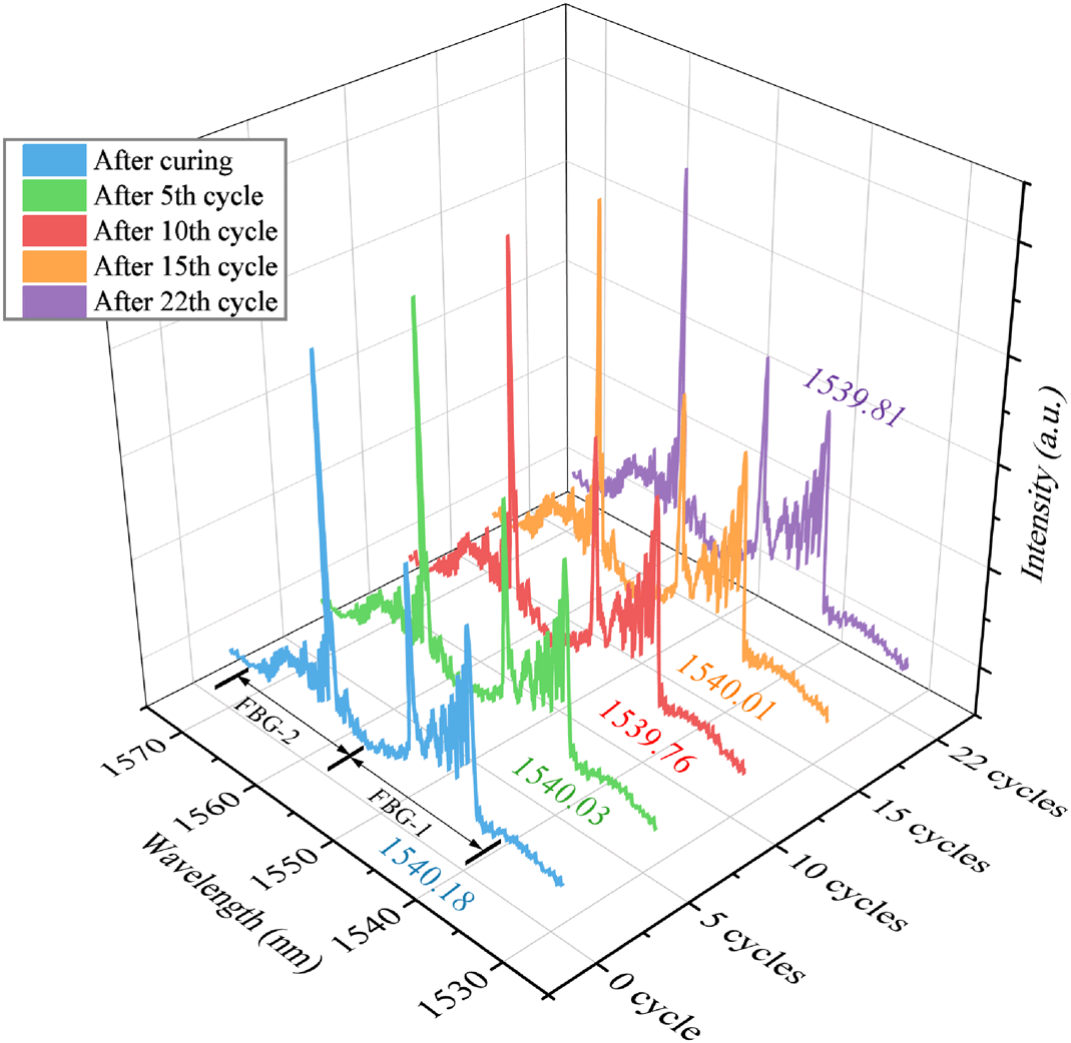
The spectra and central wavelength of FBG after thermal cycling aging.

The spectra of FBG-1 and FBG-2 at each stage of 22 thermal cycles were extracted as shown in Fig. 12. The Bragg wavelength of FBG-2 were almost the same wavelength around 1555.40 nm (± 0.05 nm), indicating that the ultimate temperature of each thermal cycle was constant nearby 100 °C (± 2 °C). The FWHM of the right peak of FBG-1 changed from 4.02 to 4.18 nm after 22 thermal cycles, representing the strain increased marginally from 3,500 to 3,700 με, which was in good accordance with the strain demodulated by temperature compensation method, and the fitted central wavelength (illustrated in Fig. 12) showed little deviation. Both these results proved that the residual stress maintained a stable value and there was no strain relaxation in glass–ceramics. Consequently, the mechanical robusticity and hermetic reliability of glass–ceramics sustained after several extreme temperature change in nuclear reactors, validated by the chirped spectra of embedded length-mismatched FBG-1.

### Results of SEM and leakage rate detecting

**Figure 13:**
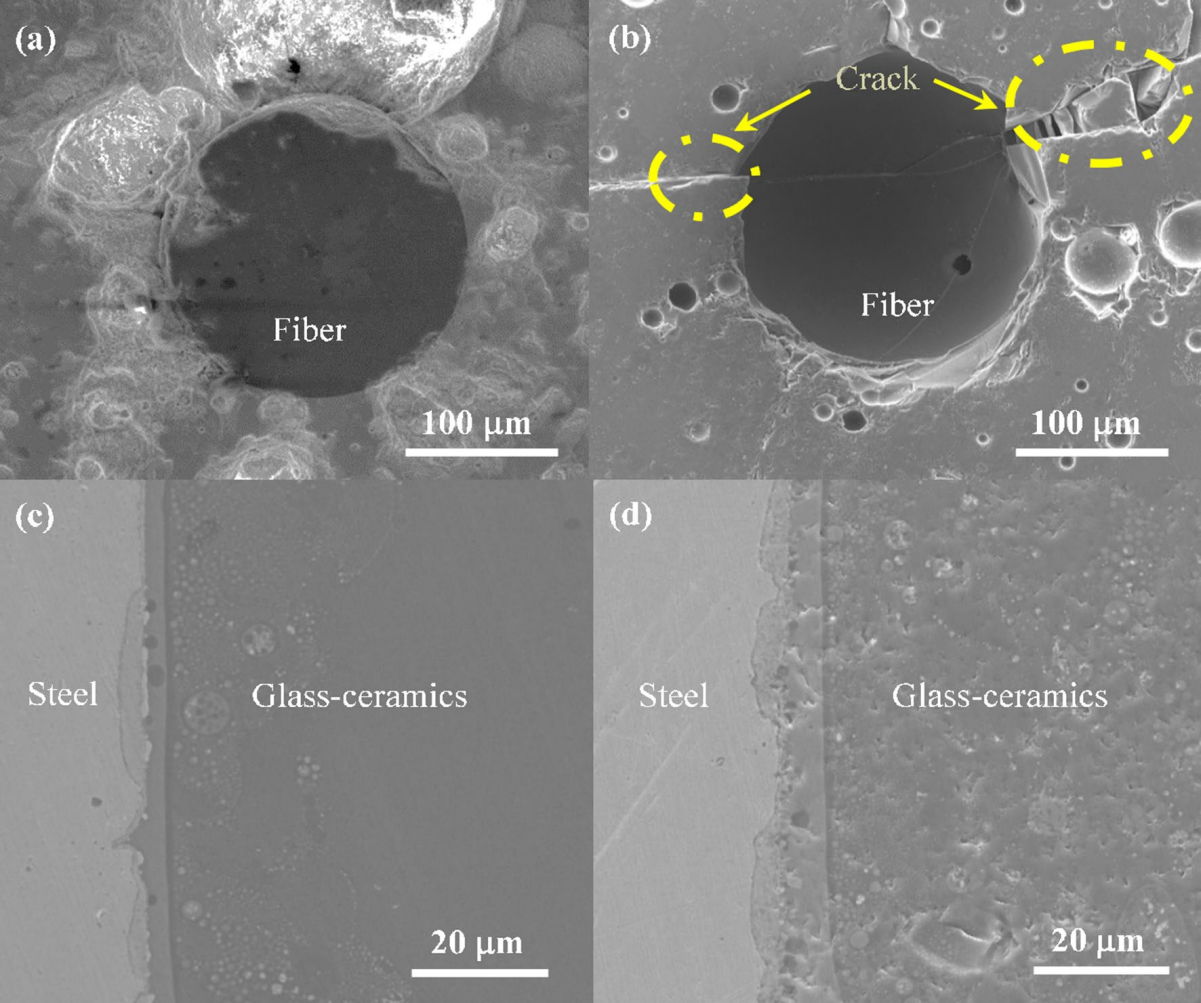
(**a**) Embedded optical fiber in glass–ceramics before thermal cycling aging; (**b**) embedded optical fiber in glass–ceramics after thermal cycling aging; (**c**) interface between glass–ceramics and steel shell before thermal cycling aging; (**d**) interface between glass–ceramics and steel shell after thermal cycling aging.

The microstructure images of EPA cross-section before and after thermal cycling aging were respectively shown in Fig. 13. The interface between fiber and glass–ceramics showed no gaps (Fig. 13a), because the constituents of them were mainly SiO_2_ and they could be fused together easily. After thermal cycling aging, the appearance of fiber was almost intact with insignificant defects generated on the interface (Fig. 13b). This guaranteed the measuring accuracy of residual strain in glass–ceramics. It was observed that some pores developed after 22 thermal cycles, causing the glass–ceramics turn from smooth to uneven (see Fig. 13c,d). The microstructure of the cross section was preserved and bonded.

**Figure 14:**
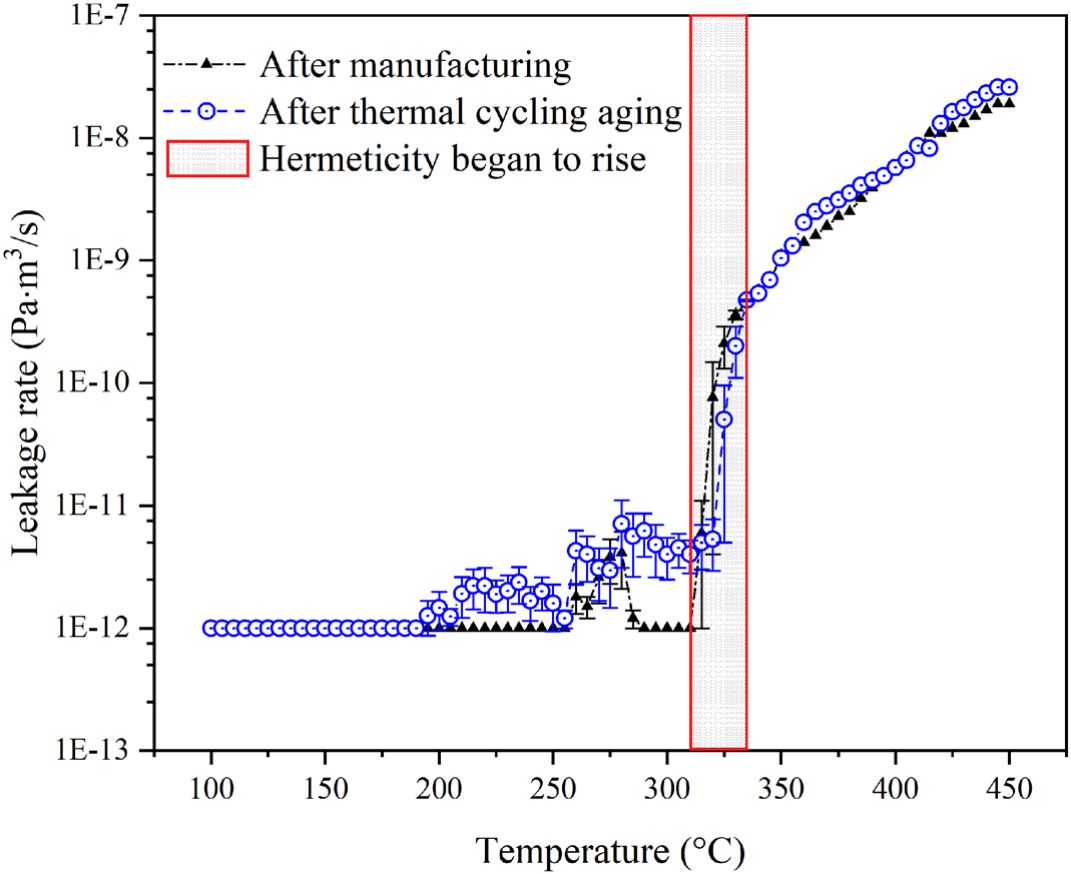
The leakage rate of MTGC-EPA model tested before and after the thermal cycling aging.

The leakage rate of MTGC-EPA models was detected at vacuum helium environment from 100 to 450 °C as shown in Fig. 14. The leakage of the model before and after thermal cycling aging showed similar trend with temperature rising. The leakage rate was almost unchanged (less than 1e^−11^ Pa·m^3^/s) before the temperature reached 310 °C. The leakage rate rose sharply when the temperature was larger than 310 °C.

It appeared that the leakage rate of model increased insignificantly after thermal cycling aging, and the hermeticity was still intact (less than 1e^-7^ Pa·m^3^/s) from 100 to 450 °C, so the model was able to bear the effect caused by several severe temperature variations in nuclear reactors (100–450 °C). The rising point of leakage rate delayed slightly after thermal cycling aging, due to the incremental residual strain explained in “MTGC-EPA” section.

## Conclusion and future work

This study investigated the on-line monitoring of residual strain in glass–ceramics based on femto-laser inscribed FBGs during thermal cycling aging experiment to verify the potential of the proposed method applied in the cycling conditions of nuclear reactors (start-up, operation, shut-down, etc.).

1. The MTGC-EPA with embedded FBG sensors was successfully sustained after 22 thermal cycles. The residual strain in glass–ceramics increased tardily as the thermal cycles went further, and the faster non-homogeneous cooling rate gently enlarged the residual strain.
2. The experimental results analysed by spectrum of the embedded length-mismatched FBG were consistent with the numerical results by combination of FEM and TMM. The period of embedded FBG was chirped by the non-uniform strain and its spectrum was divided into two sections. One was the strain-induced chirped section, and the other was the single peak effected only by temperature. This phenomenon was potential to be a novel demodulation method of temperature and strain by single embedded FBG with length mismatched grating. The bandwidth of spectrum during the thermal cycling aging broadened slightly, the trend of which was consistent with the results of temperature compensation method, indicating there were no stress relaxation happening in the glass–ceramics and the structural strength was maintained.
3. The interfaces of FBG/glass–ceramics and steel/glass–ceramics were well-preserved and bonded after thermal cycling aging. The leakage rate measured before and after thermal cycling aging showed consistent results with temperature rising, and the hermeticity of MTGC-EPA model after thermal cycling aging was proved to be intact from 100 to 450 °C.

The structural strength and hermeticity of MTGC-EPA were demonstrated to be reliable after experiencing extreme temperature variations. The methodology proposed by FBG was feasible to accomplish the real-time SHM of MTGC-EPA during the service in nuclear reactors. In the future, this method was planned to monitor the crack-induced large gradient non-uniform strain variation^31,32^ in the metal-to-glass structure through the spectrum responses.

## References

[1] Ahn J (2015). Reflections on the Fukushima Daiichi Nuclear Accident.

[2] Shekoofa O, Wang J, Qi J, Zhang J, Yin Z (2014). Analysis of residual stress for mismatch metal–glass seals in solar evacuated tubes. Sol. Energy Mater. Sol. Cells.

[3] Staff MT, Fernie JA, Mallinson PM, Whiting MJ, Yeomans JA (2016). Fabrication of a glass-ceramic-to-metal seal between Ti–6Al–4V and a strontium boroaluminate glass. Int. J. Appl. Ceram. Technol..

[4] Donald IW, Mallinson PM, Metcalfe BL, Gerrard LA, Fernie JA (2011). Recent developments in the preparation, characterization and applications of glass- and glass-ceramic-to-metal seals and coatings. J. Mater. Sci..

[5] James AW (1980). Glasses and glass ceramics for sealing to aluminum alloys ☆. J. Non-Cryst. Solids.

[6] Serbena FC, Zanotto ED (2012). Internal residual stresses in glass-ceramics: a review. J. Non-Cryst. Solids.

[7] Lacondemine T (2019). Direct observation of the displacement field and microcracking in a glass by means of X-ray tomography during in situ Vickers indentation experiment. Acta Mater..

[8] Buchheit, T. E. *et al.* Stress Mapping in Glass-to-Metal Seals using Indentation Crack Length Measurements. (Sandia National Lab.(SNL-NM), Albuquerque, NM (United States), 2017).

[9] Huntley, E., Strong, K., Elisberg, B., Meserole, S. & Diebold, T. W. Photoluminescence Spectroscopy to Determine Residual Stresses in Glass-to-Metal Seals. (Sandia National Lab.(SNL-NM), Albuquerque, NM (United States), 2019).

[10] Rajan G (2019). Polymerisation shrinkage profiling of dental composites using optical fibre sensing and their correlation with degree of conversion and curing rate. Sci. Rep..

[11] Villatoro J (2017). Accurate strain sensing based on super-mode interference in strongly coupled multi-core optical fibres. Sci. Rep..

[12] Yan A (2017). Distributed optical fiber sensors with ultrafast laser enhanced Rayleigh backscattering profiles for real-time monitoring of solid oxide fuel cell operations. Sci. Rep..

[13] Fan Z (2018). Analysis of residual stress in electrical penetration assembly based on a fiber bragg grating sensor. Sensors.

[14] Fan Z (2019). On-line monitoring of sealing glass in electrical penetration assembly based on femto-laser inscribed fiber Bragg grating sensors. Opt. Express.

[15] Mihailov SJ (2012). Fiber Bragg grating sensors for harsh environments. Sensors.

[16] Van Lancker B, Dispersyn J, De Corte W, Belis J (2016). Durability of adhesive glass-metal connections for structural applications. Eng. Struct..

[17] Li R, Tan Y, Chen Y, Hong L, Zhou Z (2019). Investigation of sensitivity enhancing and temperature compensation for fiber Bragg grating (FBG)-based strain sensor. Opt. Fiber Technol..

[18] Zhao X (2020). Ultra-high sensitivity and temperature-compensated Fabry–Perot strain sensor based on tapered FBG. Opt. Laser Technol..

[19] Lei DQ, Fu XQ, Ren YC, Yao FY, Wang ZF (2019). Temperature and thermal stress analysis of parabolic trough receivers. Renew. Energy.

[20] Dai S, Elisberg B, Calderone J, Lyon N (2017). Sealing glass-ceramics with near-linear thermal strain, part III: stress modeling of strain and strain rate matched glass-ceramic to metal seals. J. Am. Ceram. Soc..

[21] Grobnic D, Smelser CW, Mihailov SJ, Walker RB (2006). Long-term thermal stability tests at 1000 °C of silica fibre Bragg gratings made with ultrafast laser radiation. Meas. Sci. Technol..

[22] Kampling, M., Dachraoui, H., Gong, X. & Flehr, R. *Second generation fs-laser-written fiber Bragg gratings for high accuracy temperature measurement in harsh environments*. Vol. 10654 SIC (SPIE, 2018).

[23] Sampath U, Kim D, Kim H, Song M (2018). Polymer-coated FBG sensor for simultaneous temperature and strain monitoring in composite materials under cryogenic conditions. Appl. Opt..

[24] Palaniappan J (2008). Disbond growth detection in composite–composite single-lap joints using chirped FBG sensors. Compos. Sci. Technol..

[25] Caucheteur C, Lhommé F, Chah K, Blondel M, Mégret P (2006). Simultaneous strain and temperature sensor based on the numerical reconstruction of polarization maintaining fiber Bragg gratings. Opt. Lasers Eng..

[26] Diaz CA (2017). Liquid level measurement based on FBG-embedded diaphragms with temperature compensation. IEEE Sens. J..

[27] Peters K, Pattis P, Botsis J, Giaccari P (2000). Experimental verification of response of embedded optical fiber Bragg grating sensors in non-homogeneous strain fields. Opt. Lasers Eng..

[28] Prabhugoud M, Peters K (2004). Modified transfer matrix formulation for Bragg grating strain sensors. J. Lightw. Technol..

[29] Hyun-Wook L, Hyoung-Jun P, June-Ho L, Minho S (2007). Accuracy improvement in peak positioning of spectrally distorted fiber Bragg grating sensors by Gaussian curve fitting. Appl. Opt..

[30] Leal-Junior AG, Díaz C, Marques C, Frizera A, Pontes MJ (2019). 3D-Printing techniques on the development of multiparameter sensors using one FBG. Sensors.

[31] Hu H, Li S, Wang J, Wang Y, Zu L (2016). FBG-based real-time evaluation of transverse cracking in cross-ply laminates. Compos. Struct..

[32] Rajabzadeh A, Heusdens R, Hendriks RC, Groves RM (2019). Characterisation of transverse matrix cracks in composite materials using fibre Bragg grating sensors. J. Lightw. Technol..

